# Activation of α-, β-, γ- δ-, ζ- and η- class of carbonic anhydrases with amines and amino acids: a review

**DOI:** 10.1080/14756366.2019.1664501

**Published:** 2019-09-17

**Authors:** Suleyman Akocak, Claudiu T. Supuran

**Affiliations:** a Department of Pharmaceutical Chemistry, Faculty of Pharmacy, Adiyaman University, Adiyaman, Turkey;; b Dipartimento Neurofarba, Sezione di Scienze Farmaceutiche e Nutraceutiche, Università degli Studi di Firenze, Sesto Fiorentino (Florence), Italy

**Keywords:** Carbonic anhydrase, activator, isoforms, neurodegenerative, proton shuttle

## Abstract

Eight genetically distinct carbonic anhydrase (EC 4.2.1.1) enzyme families (α-, β-, γ- δ-, ζ-, η-, θ- and ι-CAs) were described to date. On the other hand, 16 mammalian α-CA isoforms are known to be involved in many diseases such as glaucoma, edema, epilepsy, obesity, hypoxic tumors, neuropathic pain, arthritis, neurodegeneration, etc. Although CA inhibitors were investigated for the management of a variety of such disorders, the activators just started to be investigated in detail for their in vivo effects. This review summarizes the activation profiles of α-, β, γ-, δ-, ζ- and η- CAs from various organisms (animals, fungi, protozoan, bacteria and archaea) with the most investigated classes of activators, the amines and the amino acids.

## 1. Introduction

Carbonic anhydrases (CAs; EC 4.2.1.1) are metalloproteins present virtually in all living organisms. CA enzymatic activity was first observed in the early 1930s, when experiments performed with hemolyzed blood samples have demonstrated that the rate of carbon dioxide release from the hemolyzed blood was higher than expected, indicating that blood could contain a catalyst for the dehydration of bicarbonate, which allows the formation of CO_2_ [1]. This catalyst, named carbonic anhydrase, was thereafter extracted from erythrocytes in 1933 by Meldrum and Roughton [2]. Upon the discovery in 1940 that zinc ions are an intrinsic cofactor of the protein, CA became the first recognized metalloenzyme. This enzyme efficiently catalyzes the reversible hydration of carbon dioxide (CO_2_) to yield bicarbonate (HCO_3_^-^) and protons (H^+^) [2,3].

$${\text{CO}}_{\text{2}}+{\text{ H}}_{\text{2}}\text{O}\;⇌\;{\text{HCO}}_{\text{3}}{}^{-}+{\text{ H}}^{+}$$

It has been known since the 1940s that CA is ubiquitous in plants [4], where it performs an essential role in CO_2_ fixation [5]. CAs, under the form of many enzyme families and isoforms, are virtually found in all living organisms, from the unicellular ones to higher vertebrates including humans. Their structure is encoded by eight evolutionary unrelated gene families, leading thus to the α-, β-, γ-, δ-, ζ-, η-, Θ-, and ι-CA classes [6–13]:

- α-CAs are Zn^2+^ metalloproteins expressed in animals, vertebrates, prokaryotes, fungi, algae, protozoa and plants [9].
- β-CAs are Zn^2+^ metalloproteins present in bacteria, plants, fungi, chloroplasts of mon-/dicotyledons [6].
- γ-CAs are Zn^2+^ or Fe, Co metalloproteins present in some plants, fungi, bacteria and archarea [6].
- δ-CAs are Co metalloproteins present in marine diatoms [7,10].
- ζ-CAs are Cd or Zn metalloproteins identified only in some marine diatoms [11].
- η-CA are Zn metalloproteins identified in Plasmodium spp. [12].
- Θ-CA are Zn metalloproteins identified in Marine diatoms [11].
- ι-CAs were only recently reported to be present in diatoms and bacteria and seem to be Mn(II) proteins [13].

CA inhibitors (CAIs) targeting mammalian CAs, are in clinical use as diuretics, antiglaucoma, antiepileptic or antiobesity agents for decades [3,6,14–18]. These diverse applications are due to the fact that at least 15 different α-CA isoforms are present in humans, being involved in critical physiological and pathological processes [14–18].

**Figure 1.**
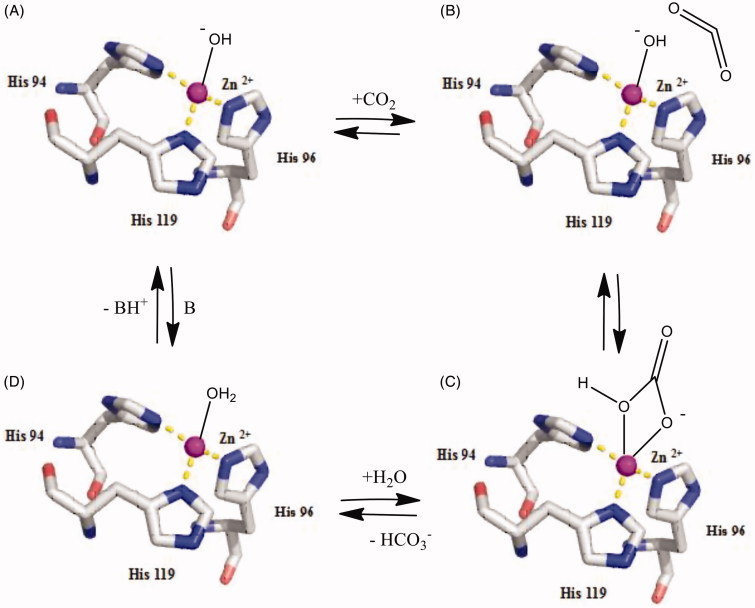
Catalytic mechanism of α-CAs [3]. A. The zinc hydroxide form of the enzyme. B. The bucleophilic attack on CO2 bound in the hydrophobic pocket. C. Bicarbonate bound to the active site metal ion. D. Acidic form of the enzyme. B in the last step of the cycle is a buffer molecule or the imidazole moiety of a His64 residue from the enzyme active site, acting as proton shuttle.

In the current review, we focused our attention on recent activation studies on α-, β-, γ-, δ-, ζ-, and η-CA classes which were explored with at least two classes of modulators of activity, amines and amino acids. The catalytic mechanism of these enzymes is in fact well understood [3]. A metal hydroxide species present in the active site of these enzymes as the fourth ligand (Figure 1(A,B)) acts as a strong nucleophile (at physiologic pH) converting the CO_2_ to bicarbonate, bis-coordinated to Zn(II), in a trigonal bipyramidal geometry (Figure 1(C)). This adduct is not very stable and reaction with a water molecule leads to liberation of bicarbonate in solution and generation of an acidic form of the enzyme incorporating a M^2+^(OH_2_) species at the metal center, which is catalytically ineffective for the hydration of CO_2_ (Figure 1(D)). In order to generate the nucleophilic, M ^2+^(OH^_^) species, a proton transfer reaction occurs, which is rate determining for the catalytic cycle in many of these quite rapid enzymes. CA enzymes typically use a metal ion (Zn^2+^ in α-, β- and γ-CAs, Fe^2+^/Co^2+^/Zn^2+^) which favors in the reduction pKa of H_2_O from 14 to 7 [6–8]. Human CAs use a Zn^2+^ ion to decrease the pKa of H_2_O bound with Zn^2+^ ion which also binds to histidine residues (His94, His96 and His119). For many α-CAs this step is assisted by a proton shuttle residue, which is His64 in most mammalian isoforms. Possessing a flexible orientation, inwards (the *in* conformation) or outwards (the *out* conformation) the zinc ion center, the imidazole moiety of this histidine, with a pKa of 6.0–7.5 is an appropriate proton shuttling residue and crucially important for the entire catalytic cycle. The process can be also assisted by endogenous molecules, which bind within the enzyme active site (as proven by X-ray crystallography and other techniques) which have been termed CA activators (CAAs) [19]. They facilitate the proton transfer reactions between the metal ion center and the external medium. It was understood that CA activators act by speeding up the deprotonation of zinc-bound water (the rate-determining step, Equation (2) in the catalytic mechanism) [19–21], with the generation of the active form of the enzyme [22] (see equations below):

(1) EZn2+−OH−+CO2⇌EZn2+−HCO3−⇌+H2OEZn2−OH2+HCO3−   

(2) EZn2+−OH2 ⇌ EZn2+−OH−+H+     −rate determining step−   

In the presence of an activator ‘A’, Equation (2) becomes (3):

(3) $${\text{EZn}}^{\text{2}+}-{\text{OH}}_{\text{2}}+\text{ A}⇌[{\text{EZn}}^{\text{2}+}-{\text{OH}}_{\text{2}}-\text{ A}]\;⇌\;[{\text{EZn}}^{\text{2}+}-{\text{HO}}^{-}-{\text{ AH}}^{+}]\;⇌\;{\text{EZn}}^{\text{2}+}-{\text{HO}}^{-}+{\text{ AH}}^{+}$$

enzyme - activator complexes

CAAs may have pharmacologic applications, activation of the mammalian enzymes was shown to enhance cognition and memory in experimental animals [23], likewise its inhibition has an opposite effect [24].

**Chart 1.**
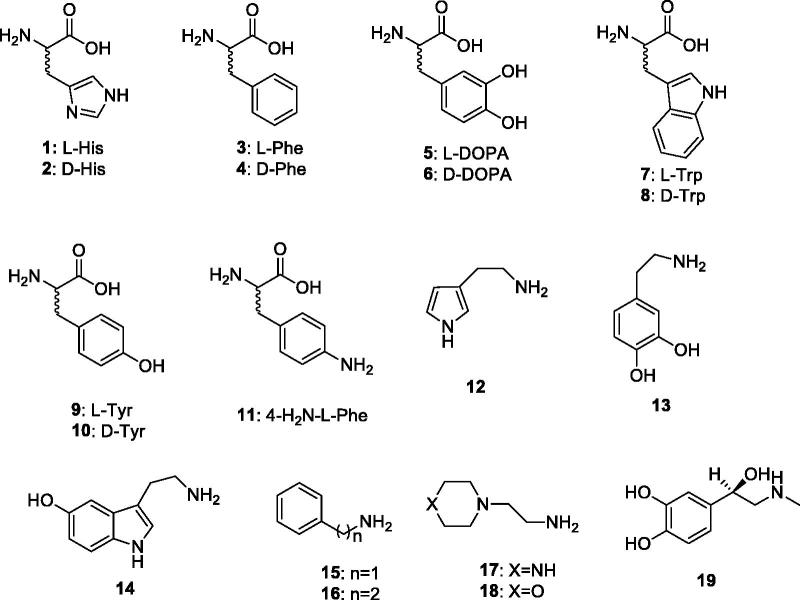
Amino acids **1–11** and amines **12–19**.

In order to better understand the catalytic mechanism of CAs belonging to the β-, δ- γ-, δ- ζ-, η-CA and Θ-CA classes, it is of crucial importance to see if these enzymes act, similar to the α-CAs, which can be activated by compounds that shuttle protons between the active site and the environment. The activation of CAs from pathogenic bacteria may be relevant for understanding the factors governing virulence and colonization of the host, because pH in the tissues surrounding the pathogens likely plays a key role in such processes and many compounds that are CAAs (biogenic amines and amino acid derivatives) are abundant in such tissues. In this review, we have carefully analyzed the activation potential of different natural, non natural, aromatic/heterocyclic amino acids and amines (compounds **1–19**) across 6 different families of CAs that were investigated based on the existing literature (Chart 1) [19–24]. These compounds have functional groups similar to their endogenous proton shuttlers, and can participate in proton transfer processes during the catalytic cycle. This study is relevant as no X-ray crystal structures of enzyme activator complexes have been reported so far for β- γ-, δ-, ζ-, η-CA and Θ-CAs.

## 2. Activation of α-CAs with amino acids and amines

Activation of the twelve catalytically active human (h) or murine (m) CA isoforms, hCA I, hCA II, hCA III, hCA IV, hCA VA, hCA VB, hCA VII, hCA IX, hCA XII, mCA XIII, hCA XIV and mCA XV with amino acids and amines (**1–19**) has been investigated by stopped flow CO_2_ hydrase assay method and are shown in Table 1 [25–29]. This bioassay is in excellent agreement with results from native mass spectrometry [30]. The following structure-activity relationship (SAR) can be summarized from data presented in Table 1 based on the activation profile of these derivatives.

**Table 1.** *In vitro* hCA I [25], hCA II [25], hCA III [26], hCA IV [26], hCA VA [27], hCA VB [27], hCA VII [28], hCA IX [29], hCA XII [29], mCA XIII [25], hCA XIV [28] and mCA XV [30] activation data with amines and amino acids (**1–19**) by a stopped-flow CO_2_ hydrase assay.

		K_A_ (µM)^a^											
No	Compound	hCA I	hCA II	hCA III	hCA IV	hCA VA	hCA VB	hCA VII	hCA IX	hCA XII	mCA XIII	hCA XIV	mCA XV
1	L-His	0.03	10.9	35.9	7.30	1.34	0.97	0.92	9.71	37.5	0.13	0.90	32.1
**2**	D-His	0.09	43	1.13	12.3	0.12	4.38	0.71	12.5	24.7	0.090	2.37	14.1
**3**	L-Phe	0.07	0.013	34.7	36.3	9.81	10.45	10.93	16.3	1.38	1.02	0.24	33.4
**4**	D-Phe	86	0.035	15.4	49.3	4.63	0.072	9.74	9.30	0.37	0.051	7.21	9.5
**5**	L-DOPA	3.1	11.4	13.5	15.3	0.036	0.063	58.3	51.3	1.67	43	12.1	6.5
**6**	D-DOPA	4.9	7.8	28.7	34.7	4.59	3.71	34.7	54.7	0.89	0.73	36.8	4.0
**7**	L-Trp	44	27	20.5	37.1	1.13	0.89	57.5	37.5	26.0	16	16.5	13.5
**8**	D-Trp	41	12	19.0	39.6	1.24	1.35	39.6	43.6	28.1	0.81	18.0	8.7
**9**	L-Tyr	0.02	0.011	34.1	25.1	2.45	0.044	20.3	25.3	25.8	–	21.8	8.9
**10**	D-Tyr	0.04	0.013	–	–	–	–	–	–	–	–	–	–
**11**	4-H_2_N-L-Phe	0.24	0.15	43.2	0.079	2.76	2.17	18.7	48.7	1.09	–	2.90	16.3
**12**	Histamine	2.1	125	36.9	25.3	0.010	3.52	37.5	35.1	27.9	4.6	0.010	18.5
**13**	Dopamine	13.5	9.2	33.2	30.9	0.13	7.85	0.89	0.92	0.67	27	14.6	7.1
**14**	Serotonin	45	50	0.78	3.14	6.33	0.11	0.93	33.1	0.30	0.51	6.5	7.5
**15**	2-Pyridyl-methylamine	26	34	1.03	5.19	23.56	0.24	43.7	1.07	41.5	3.8	21.7	11.6
**16**	2–(2-Aminoethyl)pyridine	13	15	1.10	7.13	7.62	0.094	27.8	0.013	0.69	46	6.9	11.9
**17**	1–(2-Aminoethyl)-piperazine	7.4	2.3	0.32	24.9	6.04	0.91	32.5	0.009	48.3	54	18.3	10.4
**18**	4–(2-Aminoethyl)morpholine	0.14	0.19	0.091	1.30	0.089	1.15	64.3	0.43	0.24	0.013	5.4	9.3
**19**	L-Adrenaline	0.09	96	36.4	45.0	–	–	–	60	0.87	–	36.1	6.9

a Mean from 3 different determinations (errors in the range of 5–10% of the reported values.

Compounds **1–19** generally activated, these CA isoforms in a very different manner based on their structures. Nanomolar potencies were observed for several isozymes. For example, hCA I was activated by compounds **1** (L-His), **2** (D-His), **3** (L-His), **9** (L-Tyr), **10** (D-Tyr), and **19** (L-adrenaline) with K_A_s ranging from 20 to 90 nM. The best activation profile was observed against one of the most abundant cytosolic isoform hCA II with K_A_s ranging from 125 µM to 11 nM. Specifically, compounds **3** (L-His), **9** (L-Tyr), and **10** (D-Tyr) showed good activation potency with K_A_s of 13, 11 and 13 nM, respectively. Other cytosolic isoforms hCA III and hCA VII were weakly activated, in general, by these series of amines and amino acids **1–19**. The remaining cytosolic isoform mCA XIII was moderately activated by most of the compounds with K_A_s ranging from 0.24 to 48.3 µM. Among the mitochondrial isoforms hCA VB was slightly better activated than hCA VA by these amines and amino acids. Interestingly, compound **5** (L-DOPA) showed nanomolar potency against both isozymes, hCA VA and VB, with K_A_s of 36 and 63 nM, respectively. Only one compound **11** (4-H_2_N-L-Phe) had nanomolar activity against membrane-bound isoform hCA IV with a K_A_ of 79 nM. On the other hand, another transmebrane-bound tumor overexpressed isoform hCA IX was moderately activated by most of the tested compounds, except the compound **16** and **17** which showed one of the best activation profile from the Table 1 with K_A_s of 13 and 9 nM, respectively. The CA activating effects of amines and amino acids **1–19** on the remaining membrane-bound isoforms hCA XII, hCA XIV and mCA XV were moderate to weak and most of the results were very close the each others (Table 1).

## 3. Activation of β-CAs with amino acids and amines

In literature, there are many β-CAs which were investigated in details, among which Cab (from *Methanobacterium thermoautotrophicum*), scCA (from *Saccharomyces cerevisiae*), CgCA (from *Candida glabrata*), MgCA (from *Malassezia globosa*), VchCAβ (from *Vibrio cholerae*, mtCA 3 (from *Mycobacterium tuberculosis*), BsuCA1 (from *Brucella suis*), FtuCA (from *Francisella tularensis*), LdcCA (from *Leishmania donovani chagasi*), and EhiCA (from *Entameba histolytica*). Their activation by by amines and amino acid derivatives was investigated in the last several years [31–38] – Table 2. In general, good to moderate activation effects were obtained against all β-CAs, except FtuCA, by using amino acid and amine derivatives **1–19**. Among the β-CAs, the best activation profile was observed against LdcCA, for which two compounds, **16** and **17**, showed nanomolar potency, with K_A_s of 12 and 9 nM, respectively. Interestingly, these two compounds have (2-aminoethyl) groups in their structures. Other interesting results were obtained against scCA for which the activation profile was better with amines (K_A_s: 0.95–21.3 µM) than with amino acids (K_A_s: 82–91 µM). Furthermore, VchCAβ and BsuCA1 was also activated efficiently, with K_A_s of 0.18–20.3 and 0.70–43.1 µM, by amino acids and amines, respectively. Specifically, VchCAβ was activated slightly more effectively by amines (K_A_s: 0.18–12.8 µM) than by amino acid derivatives (K_A_s: 0.94–20.3 µM). For BsuCA1 activities of most compounds are close to each other, except the compounds **2**, **8**, and **17** with K_A_s of 12.3, 13.7 and 43.1 µM, respectively, which are the least effective CAAs. In the case of FtuCA, most of the amines and amino acid derivatives (compounds **5**, **9–14**, **16**, **18** and **19**) investigated so far showed weak activation effects, with activation constants >100 µM. The remaining activators were also moderately active against FtuCA, with K_A_s ranging between 30.5 to 78.3 µM. Other β-CAs (Cab, CgCA, MgCA, mtCA 3 and EhiCA) were activated in a different manner, as seen from Table 2, with most of the activation constants in a limited range of values.

**Table 2.** *In vitro* β-CA (Cab [31], scCA [31–34], CgCA [34], MgCA [32], VchCAβ [35], mtCA 3 [36], BsuCA1 [37], FtuCA [37], LdcCA [38, 39], and EhiCA [38]) activation data with amines and amino acids (**1–19**).

		K_A_ (µM)^a^									
No	Compound	Cab	scCA	CgCA	MgCA	VchCAβ	mtCA 3	BsuCA1	FtuCA	LdcCA	EhiCA
**1**	L-His	69	82	37	29.3	20.3	18.2	1.76	40.7	8.21	78.7
**2**	D-His	57	85	21.2	18.1	18.0	32.5	12.3	78.3	4.13	9.83
**3**	L-Phe	70	86	24.1	34.1	15.4	30.6	1.16	69.1	9.16	16.5
**4**	D-Phe	10.3	86	15.7	10.7	5.12	44.1	1.21	75.0	3.95	10.1
**5**	L-DOPA	11.4	90	23.3	8.31	8.36	30.0	2.07	>100	1.64	16.6
**6**	D-DOPA	15.6	89	15.1	13.7	6.27	9.74	2.34	44.8	5.47	4.05
**7**	L-Trp	16.9	91	22.8	10.1	4.18	8.98	1.25	34.1	4.02	5.24
**8**	D-Trp	41	90	12.1	12.5	5.89	43.7	13.7	30.5	6.18	4.95
**9**	L-Tyr	10.5	85	9.5	15.7	6.15	28.9	1.38	>100	8.05	4.52
**10**	D-Tyr	19.2	84	7.1	25.1	0.94	17.6	0.95	>100	1.27	1.07
**11**	4-H_2_N-L-Phe	89	21.3	31.6	13.4	7.21	40.5	1.18	>100	15.9	8.12
**12**	Histamine	76	20.4	27.4	10.9	9.50	34.2	3.71	>100	0.74	7.38
**13**	Dopamine	51	13.1	27.6	9.43	1.24	12.1	1.54	>100	0.81	30.8
**14**	Serotonin	62	15.0	16.7	14.2	1.37	10.3	4.26	>100	0.62	4.94
**15**	2-Pyridyl-methylamine	18.7	16.2	15.0	6.12	0.18	43.3	1.62	46.3	0.23	>100
**16**	2–(2-Aminoethyl)pyridine	40	11.2	16.3	7.30	1.00	45.9	5.20	>100	0.012	>100
**17**	1–(2-Aminoethyl)-piperazine	13.8	9.3	14.9	0.81	0.24	50.3	43.1	51.8	0.009	43.8
**18**	4–(2-Aminoethyl)morpholine	18.5	10.2	10.1	5.82	12.8	52.0	9.56	>100	0.94	>100
**19**	L-Adrenaline	11.5	0.95	10.8	0.72	8.73	52.2	0.70	>100	4.89	25.6

a Mean from 3 different determinations (errors in the range of 5–10% of the reported values, data not shown).

## 4. Activation of γ-, δ-, ζ-, and η-CAs with amino acids and amines

Activation studies were also performed recently against γ-CAs, such as Zn-Cam and Co-Cam (from the Archaeon *Methanosarcina thermpophila*), BpsγCA (from the pathogenic bacterium *Burkhalderia pseudomallei*), PhaCA (from the cyanobacterium *Pseudoalteromonas haloplanktis*), and CpsCA (from another cyanobacteriu, *Colwellia psychrerythraea*), as well as δ-CAs, such as TweCAδ (from the diatom *Thalassiosira weissflogii*)], ζ-CA, such as ZnTweCAζ (from the same diatom, *Thalassiosira weissflogii*)], and η-CAs, such as PfaCA (from *Plasmodium falciparum*) [31, 40–44]. Among them, an interesting activation profile was observed for some of the γ- class CAs, such as BpsγCA. Most of the tested compounds showed nanomolar potency against this enzyme. Specifically, BpsγCA was efficiently activated by compounds **2**, **5**, **8**, **11**, **13**, and **16–19** with activation constants ranging between 9 to 86 nM. Interestingly, the ζ- class CA, ZnTweCAζ was activated slightly more efficiently by amines (K_A_s of 92 nM to 10.1 µM) than by amino acids (K_A_s of 0.62 to 15.4 µM), which is just the opposite in the case of the η- class CA PfaCA, for which K_A_s ranging from 0.12 to 8.55 µM were obtained for amino acid derivatives and between 0.71 and 9.97 µM for amines (Table 3). A wide range of activities of the various activators for the remaining CAs was observed, such as for γ- class of CAs, Co-Cam and PhaCA, which were moderately activated by amino acid derivatives and amines with K_A_s of 0.72–135 µM (Table 3). Other γ-CAs, such as Zn-Cam and CpsCA were less prone to be activated, as compared to other γ- CAs investigated so far, with activation constants ranging between 4.79 to >100 µM. The unique δ- class CA investigated in details at this moment, TweCAδ, was efficiently activated by most of the amino acid derivatives and amines *1–19*, with K_A_s ranging between 51 nM and 18.9 µM.

**Table 3.** *In vitro* γ-CA (Zn-Cam [31], Co-Cam [31], BpsγCA [40], PhaCA [41], and CpsCA [41]), δ-CA (TweCAδ) [42], ζ-CA (ZnTweCAζ) [43], and η-CA (PfaCA) [44] activation data with amines and amino acids (**1–19**).

		K_A_ (µM)^a^							
No	Compound	Zn-Cam	Co-Cam	BpsγCA	PhaCA	CpsCA	TweCAδ	ZnTweCAζ	PfaCA
**1**	L-His	68	135	24.7	12.6	47.5	0.75	0.81	1.06
**2**	D-His	46	73	0.086	9.41	35.9	4.90	7.16	2.19
**3**	L-Phe	68	70	1.73	15.8	>100	2.15	15.4	0.43
**4**	D-Phe	42	24	0.13	3.19	15.4	1.96	9.63	0.75
**5**	L-DOPA	39	38	0.072	1.08	4.79	2.11	3.19	0.12
**6**	D-DOPA	37	41	0.98	0.72	11.2	6.24	2.87	0.39
**7**	L-Trp	38	47	0.43	7.12	21.3	0.93	8.54	5.21
**8**	D-Trp	33	68	0.052	13.9	36.8	0.69	1.79	8.47
**9**	L-Tyr	24	53	0.20	1.02	19.5	1.52	0.98	1.02
**10**	D-Tyr	–	–	32.8	7.35	18.4	0.051	0.62	8.55
**11**	4-H_2_N-L-Phe	72	22	0.009	3.27	17.2	18.9	7.90	1.00
**12**	Histamine	63	9.2	0.12	6.48	20.6	1.34	1.27	9.86
**13**	Dopamine	54	18.4	0.014	8.70	32.1	0.51	10.1	9.97
**14**	Serotonin	38	0.97	0.10	9.05	34.8	0.90	3.06	7.18
**15**	2-Pyridyl-methylamine	11.4	8.7	2.36	2.39	21.5	5.28	0.88	3.69
**16**	2–(2-Aminoethyl)pyridine	24	18.5	0.034	18.7	38.2	8.16	0.85	6.75
**17**	1–(2-Aminoethyl)-piperazine	10.1	16.1	0.018	15.1	33.0	4.37	0.12	0.71
**18**	4–(2-Aminoethyl)morpholine	45	38	0.015	10.1	34.2	7.39	0.15	5.33
**19**	L-Adrenaline	39	8.9	0.019	17.5	79.8	2.43	0.092	2.40

a Mean from 3 different determinations (errors in the range of 5–10% of the reported values, data not shown).

## 5. Conclusions and future perspective

To our knowledge, this is the first article that summarizes the activation profile of all classes of CAs (the α-, β-, γ-, δ-, ζ-, and η-CA) with a small library of amines and amino acid derivatives. This panel of investigated amino acids and amines showed considerable activating properties, with a well defined structure–activity relationship, but without net differences between the various CA families. Even if the available activators are not isoform-selective (for the many α-CAs of human or other origins), as already mentioned above, in the last period, their possible use as pharmacological agents for memory therapy or for artificial tissues engineering started to be explored [23,24], with very promising results being obtained. There is however a stringent need for having more effective, isoform-selective and possibly non-autacoid or amino acid derived compounds, which may possess a rather complicated polypharmacology [3]. Furthemore, the investigations of the activating effects of non-human CAs are still in their infancy, with very few *in vitro* studies being available on the non-α-CA activators. Indeed, only in the few several years the first activation studies of β-, γ-, δ-, ζ-, and η-CAs from various organisms have been reported, which allowed the identification of compounds active in the nanomolar to micromolar range. However, no drug design studies of CAAs targeting these enzymes were performed so far, which is one of the future objectives of research in this area. In addition, almost nothing is known regarding the *in vivo* effects of CAAs in organisms other than the vertebrates (human and rodents). As briefly mentioned, many pathogenic bacteria, fungi or protozoans live in various niches which are potentially rich in endogenous activators of the amine and amino acid type. A deep understanding of the role that these modulators of activity may play in the interaction between the host and the pathogen, may lead to relevant biomedical discoveries, but this is an entire new field to be explored in the future.

## References

[1] Van Slyke DD, Hawkins JA Studies of gas and electrolyte equilibria in blood XVI. The evolution of carbon dioxide from blood and buffer solutions. J Biol Chem 1930;87:265–79.

[2] Meldrum NU, Roughton F Carbonic anhydrase. Its preparation and properties. J Physiol (Lond) 1933;80:113–42. 16994489 10.1113/jphysiol.1933.sp003077 PMC1394121

[3] (a) Supuran CT Carbonic anhydrase activators. Future Med Chem 2018;10:561–73. (b) Temperini C, Scozzafava A, Supuran CT Carbonic anhydrase activation and the drug design. Curr Pharm Des 2008;14:708–15. (c) Supuran CT Carbonic anhydrases: novel therapeutic applications for inhibitors and activators. Nat Rev Drug Discov 2008;7:168–81. (d) Akocak S, Ilies MA, Next-Generation primary sulfonamide carbonic anhydrase inhibitors In: Supuran C.T., Cappasso C (Eds.) Targeting carbonic anhydrases, future science, London; 2014, pp. 35–51; (e) Zamanova S, Shabana AM, Mondal UK, Ilies MA Carbonic anhydrases as disease markers. Expert Opin Ther Targets 2019;29:509–33.

[4] Bradfield JR Plant carbonic anhydrase. Nature 1947;159:467. 20295218 10.1038/159467a0

[5] (a) Capasso C, Supuran CT An overview of the alpha-, beta- and gamma-carbonic anhydrases from Bacteria: can bacterial carbonic anhydrases shed new light on evolution of bacteria? J Enzyme Inhib Med Chem 2015;30:325–32. (b) Smith KS, Jakubzick C, Whittam TS, Ferry JG Carbonic anhydrase is an ancient enzyme widespread in prokaryotes. Proc Natl Acad Sci USA 1999;96:15184–9. 10.3109/14756366.2014.910202 24766661

[6] (a) Supuran CT Applications of carbonic anhydrases inhibitors in renal and central nervous system diseases. Expert Opin Ther Pat 2018;28:713–21. (b) Supuran CT Carbonic anhydrase inhibitors and their potential in a range of therapeutic areas. Expert Opin Ther Pat 2018;28:709–12. (c) Supuran CT Carbonic anhydrase inhibitors as emerging agents for the treatment and imaging of hypoxic tumors. Expert Opin Investig Drugs 2018;27:963–70. (d) Nocentini A, Supuran CT Carbonic anhydrase inhibitors as antitumor/antimetastatic agents: a patent review (2008-2018). Expert Opin Ther Pat 2018;28:729–40. (e) Nocentini A, Supuran CT Advances in the structural annotation of human carbonic anhydrases and impact on future drug discovery. Expert Opin Drug Discov 2019;1–23. (in press).

[7] (a) Supuran CT, Capasso C The η-class carbonic anhydrases as drug targets for antimalarial agents . Expert Opin Ther Targets 2015;19:551–63. (b) Capasso C, Supuran CT Bacterial, fungal and protozoan carbonic anhydrases as drug targets. Expert Opin Ther Targets 2015;19:1689–704. (c) Supuran CT, Capasso C Biomedical applications of prokaryotic carbonic anhydrases. Expert Opin Ther Pat 2018;28:745–54.

[8] (a) Ozensoy Guler O, Capasso C, Supuran CT A magnificient enzyme superfamily: carbonic anhydrases, their purification and characterization. J Enzym Inhib Med Chem 2016;31:689–94. (b) De Simone G, Supuran CT (In)organic anions as carbonic anhydrase inhibitors. J Inorg Biochem 2012;111:117–29. (c) Supuran CT Carbonic anhydrase inhibition and the management of hypoxic tumors. Metabolites 2017;7:E48.

[9] (a) Alterio V, Esposito D, Monti SM, et al. Crystal structure of the human carbonic anhydrase II adduct with 1-(4-sulfamoylphenyl-ethyl)-2,4,6-triphenylpyridinium perchlorate, a membrane-impermeant, isoform selective inhibitor. J Enzyme Inhib Med Chem 2018;33:151–7. (b) Innocenti A, Vullo D, Scozzafava A, et al. Carbonic anhydrase inhibitors. Inhibition of mammalian isoforms I – XIV with a series of substituted phenols including paracetamol and salicylic acid. Bioorg Med Chem 2008;16:7424–8.(c) Nocentini A, Bonardi A, Gratteri P, et al. Steroids interfere with human carbonic anhydrase activity by using alternative binding mechanisms. J Enzyme Inhib Med Chem 2018;33:1453–9. (d) Tars K, Vullo D, Kazaks A, et al. Sulfocoumarins (1,2-benzoxathiine-2,2-dioxides): a class of potent and isoform-selective inhibitors of tumor-associated carbonic anhydrases. J Med Chem 2013;56:293–300.

[10] (a) Del Prete S, Vullo D, Scozzafava A, et al. Cloning, characterization and anion inhibition study of the δ-class carbonic anhydrase (TweCA) from the marine diatom Thalassiosira weissflogii. Bioorg Med Chem 2014;22:531–7. (b) Vullo D, Del Prete S, Osman SM, et al. Sulfonamide inhibition studies of the δ-carbonic anhydrase from the diatom Thalassiosira weissflogii. Bioorg Med Chem Lett 2014;24:275–9. (c) Del Prete S, Vullo D, De Luca V, et al. Biochemical characterization of the δ-carbonic anhydrase from the marine diatom *Thalassiosira weissflogii*, TweCA. J Enzyme Inhib Med Chem 2014;29:906–11.

[11] (a) Supuran CT, Capasso C An overview of the bacterial carbonic anhydrases. Metabolites 2017;7:56 (b) Clare BW, Supuran CT Carbonic anhydrase activators. 3: Structure‐activity correlations for a series of isozyme II activators. J Pharm Sci 1994;83:768–73.(c) Supuran CT Carbonic anhydrases and metabolism. Metabolites 2018;8:25 (d) Supuran CT Carbon- versus sulphur-based zinc binding groups for carbonic anhydrase inhibitors? J Enzyme Inhib Med Chem 2018;33:485–95.

[12] (a) Del Prete S, Vullo D, Fisher GM, et al. Discovery of new family of carbonic anhydrases in the malaria pathogen Plasmodium falciparum- the η-carbonic anhydrases. Bioorg Med Chem 2014;24:4389–96. (b) De Simone G, Di Fiore A, Capasso C, Supuran CT The zinc coordination pattern in the η-carbonic anhydrase from Plasmodium falciparum is different from all other carbonic anhydrase genetic families. Bioorg Med Chem Lett 2015;25:1385–9. 10.1016/j.bmcl.2014.08.015 25168745

[13] Jensen EL, Clement R, Kosta A, et al. A new widespread subclass of carbonic anhydrase in marine phytoplankton. Isme J 2019;13:2094–106. 31024153 10.1038/s41396-019-0426-8 PMC6776030

[14] (a) Alterio V, Di Fiore A, D'Ambrosio K, et al. Multiple binding modes of inhibitors to carbonic anhydrases: how to design specific drugs targeting 15 different isoforms? Chem Rev 2012;112:4421–68. (b) Briganti F, Pierattelli R, Scozzafava A, Supuran CT Carbonic anhydrase inhibitors. Part 37. Novel classes of carbonic anhydrase inhibitors and their interaction with the native and cobalt-substituted enzyme: kinetic and spectroscopic investigations. Eur J Med Chem 1996;31:1001–10. (c) Supuran CT How many carbonic anhydrase inhibition mechanisms exist? J Enzyme Inhib Med Chem 2016;31:345–60. (d) Supuran CT Advances in structure-based drug discovery of carbonic anhydrase inhibitors. Expert Opin Drug Discov 2017;12:61–88. (e) Supuran CT Structure and function of carbonic anhydrases. Biochem J 2016;473:2023–32.(f) Neri D, Supuran CT Interfering with pH regulation in tumours as a therapeutic strategy. Nat Rev Drug Discov 2011;10:767–77. (g) Supuran CT, Vullo D, Manole G, et al. Designing of novel carbonic anhydrase inhibitors and activators. Curr Med Chem Cardiovasc Hematol Agents 2004;2:49–68. (h) Akocak S, Alam MR, Shabana AM, et al. PEGylated Bis-Sulfonamide carbonic anhydrase inhibitors can efficiently control the growth of several carbonic Anhydrase IX-Expressing carcinomas. J Med Chem 2016;59:5077–88.

[15] (a) Pacchiano F, Carta F, McDonald PC, et al. Ureido-substituted benzenesulfonamides potently inhibit carbonic anhydrase IX and show antimetastatic activity in a model of breast cancer metastasis. J Med Chem 2011;54:1896–902. (b) Lou Y, McDonald PC, Oloumi A, et al. Targeting tumor hypoxia: suppression of breast tumor growth and metastasis by novel carbonic anhydrase IX inhibitors. Cancer Res 2011;71:3364–76. (c) Pacchiano F, Aggarwal M, Avvaru BS, et al. Selective hydrophobic pocket binding observed within the carbonic anhydrase II active site accommodate different 4-substituted-ureido-benzenesulfonamides and correlate to inhibitor potency. Chem Commun (Camb) 2010;46:8371–3. (d) Köhler K, Hillebrecht A, Schulze Wischeler J, et al. Saccharin inhibits carbonic anhydrases: possible explanation for its unpleasant metallic aftertaste. Angew Chem Int Ed Engl 2007;46:7697–9.

[16] (a) Krall N, Pretto F, Decurtins W, et al. A Small‐Molecule drug conjugate for the treatment of carbonic anhydrase IX expressing tumors. Angew Chem Int Ed Engl 2014;53:4231–5.(b) El-Gazzar MG, Nafie NH, Nocentini A, et al. Carbonic anhydrase inhibition with a series of novel benzenesulfonamide-triazole conjugates. J Enzyme Inhib Med Chem 2005; 20:333–40. (c) Eldehna WM, Abo-Ashour MF, Berrino E, et al. SLC-0111 enaminone analogs, 3/4-(3-aryl-3-oxopropenyl) aminobenzenesulfonamides, as novel selective subnanomolar inhibitors of the tumor-associated carbonic anhydrase isoform IX. Bioorg Chem 2019;83:549–58. (d) Abo-Ashour MF, Eldehna WM, Nocentini A, et al. Novel synthesized SLC-0111 thiazole and thiadiazole analogues: Determination of their carbonic anhydrase inhibitory activity and molecular modeling studies. Bioorg Chem 2019;87:794–802.

[17] (a) Akocak S, Lolak N, Nocentini A, et al. Synthesis and biological evaluation of novel aromatic and heterocyclic bis-sulfonamide Schiff bases as carbonic anhydrase I, II, VII and IX inhibitors. Bioorg Med Chem 2017;25:3093–7. (b) Akocak S, Lolak N, Bua S, et al. Synthesis and biological evaluation of novel N,N`-diaryl cyanoguanidines acting as potent and selective carbonic anhydrase II inhibitors. Bioorg Chem 2018;77:245–51. (c) Lolak N, Akocak S, Bua S, et al. Design and synthesis of novel 1,3-diaryltriazene-substituted sulfonamides as potent and selective carbonic anhydrase II inhibitors. Bioorg Chem 2018;77:542–7. (d) Akocak S, Lolak N, Bua S, et al. Discovery of novel 1,3-diaryltriazene sulfonamides as carbonic anhydrase I, II, VII, and IX inhibitors. J Enzyme Inhib Med Chem 2018;33:1575–80.

[18] (a) Lolak N, Akocak S, Bua S, Supuran CT Design, synthesis and biological evaluation of novel ureido benzenesulfonamides incorporating 1,3,5-triazine moieties as potent carbonic anhydrase IX inhibitors. Bioorg Chem 2019;82:117–22. (b) Lolak N, Akocak S, Bua S, et al. Discovery of new ureido benzenesulfonamides incorporating 1,3,5-triazine moieties as carbonic anhydrase I, II, IX and inhibitors. Bioorg Med Chem 2019;27:1588–94. (c) Shabana AM, Mondal UK, Alam R, et al. pH-sensitive multiligand gold nanoplatform targeting carbonic anhydrase IX enhances the delivery of Doxorubicin to hypoxic tumor spheroids and overcomes the hypoxia-induced chemoresistance. ACS Appl Mater Interfaces 2018;10:17792–808.

[19] Briganti F, Mangani S, Orioli P, et al. Carbonic anhydrase activators: X-ray crystallographic and spectroscopic investigations for the interaction of isozymes I and II with histamine. Biochemistry 1997;36:10384–92. 9265618 10.1021/bi970760v

[20] (a) Temperini C, Scozzafava A, Vullo D, Supuran CT Carbonic anhydrase activators. Activation of isozymes I, II, IV, VA, VII, and XIV with l- and d-histidine and crystallographic analysis of their adducts with isoform II: engineering proton-transfer processes within the active site of an enzyme. Chemistry 2006;12:7057–66. (b) Temperini C, Scozzafava A, Vullo D, Supuran CT Carbonic anhydrase activators. Activation of isoforms I, II, IV, VA, VII, and XIV with L- and D-phenylalanine and crystallographic analysis of their adducts with isozyme II: stereospecific recognition within the active site of an enzyme and its consequences for the drug design. J Med Chem 2006;49:3019–27. (c) Temperini C, Innocenti A, Scozzafava A, Supuran CT Carbonic anhydrase activators: kinetic and X-ray crystallographic study for the interaction of D- and L-tryptophan with the mammalian isoforms I-XIV. Bioorg Med Chem 2008;16:8373–8. (d) Temperini C, Innocenti A, Scozzafava A, et al. Carbonic anhydrase activators: L-Adrenaline plugs the active site entrance of isozyme II, activating better isoforms I, IV, VA, VII, and XIV. Bioorg Med Chem Lett 2007;17:628–35.

[21] (a) Akocak S, Lolak N, Vullo D, et al. Synthesis and biological evaluation of histamine Schiff bases as carbonic anhydrase I, II, IV, VII, and IX activators. J Enzyme Inhib Med Chem 2017;32:1305–12. (b) Akocak S, Lolak N, Bua S, et al. α-Carbonic anhydrases are strongly activated by spinaceamine derivatives. Bioorg Med Chem 2019;27:800–4. (c) Akocak S, Lolak N, Bua S, et al. Activation of human α-carbonic anhydrase isoforms I, II, IV and VII with bis-histamine Schiff bases and bis-spinaceamine substituted derivatives. J Enzyme Inhib Med Chem 2019;34:1193–8. (d) Dave K, Scozzafava A, Vullo D, et al. Pyridinium derivatives of histamine are potent activators of cytosolic carbonic anhydrase isoforms, I, II and VII. Org Biomol Chem 2011;9:2790–800. (e) Dave K, Ilies MA, Scozzafava A, et al. An inhibitor-like binding mode of a carbonic anhydrase activator within the active site of isoform II. Bioorg Med Chem Lett 2011;21:2764–8.

[22] (a) Bhatt A, Mondal UK, Supuran CT, et al. Crystal structure of Carbonic Anhydrase II in complex with an activating ligand: Implications in neuronal function. Mol Neurobiol 2018;55:7431–7. (b) Temperini C, Scozzafava A, Supuran CT Carbonic anhydrase activators: the first X-ray crystallographic study of an adduct of isoform I. Bioorg Med Chem Lett 2006;16:5152–6. (c) Temperini C, Scozzafava A, Puccetti L, Supuran CT Carbonic anhydrase activators: X-ray crystal structure of the adduct of human isozyme II with L-histidine as a platform for the design of stronger activators. Bioorg Med Chem Lett 2005;15:5136–41. (d) Draghici B, Vullo D, Akocak S, et al. Ethylene bis-imidazoles are highly potent and selective activators for isozymes VA and VII of carbonic anhydrase, with a potential nootropic effect. Chem Commun 2014;50:5980–3.

[23] (a) Canto de Souza L, Provensi G, Vullo D, et al. Carbonic anhydrase activation enhances object recognition memory in mice through phosphorylation of the extracellular signal-regulated kinase in the cortex and the hippocampus. Neuropharmacology 2017;118:148–56. (b) Sanku RKK, John JS, Ilies MA, Walker EA Potential learning and memory disruptors and enhancers in a simple, 1-day operant task in mice. Behavioural Pharmacol 2018;29:482–92. 10.1016/j.neuropharm.2017.03.009 28286213

[24] Wang X, Schröder HC, Schlossmacher U, et al. Modulation of the initial mineralization process of SaOS-2 cells by carbonic anhydrase activators and polyphosphate. Calcif Tissue Int 2014;94:495–509. 24374859 10.1007/s00223-013-9833-4

[25] Parkkila S, Vullo D, Puccetti L, et al. Carbonic anhydrase activators: Activation of isozyme XIII with amino acids and amines. Bioorg Med Chem Lett 2006;16:3955–9. 16730978 10.1016/j.bmcl.2006.05.023

[26] Vullo D, Nishimori I, Scozzafa A, Supuran CT Carbonic anhydrase activators: Activation of the human cytosolic isozyme III and membrane-associated isoform IV with amino acids and amines. Bioorg Med Chem Lett 2008;18:4303–7. 18627905 10.1016/j.bmcl.2008.06.075

[27] Vullo D, Nishimori I, Innocenti A, et al. Carbonic anhydrase activators: An activation study of the human mitochondrial isoforms VA and VB with amino acids and amines. Bioorg Med Chem Lett 2007;17:1336–40. 17174092 10.1016/j.bmcl.2006.11.075

[28] Vullo D, Innocenti A, Nishimori I, et al. Carbonic anhydrase activators: Activation of the human isoforms VII (cytosolic) and XIV (transmembrane) with amino acids and amines. Bioorg Med Chem Lett 2007;17:4107–12. 17540561 10.1016/j.bmcl.2007.05.052

[29] (a) Pastorekova S, Vullo D, Nishimori I, et al. Carbonic anhydrase activators: Activation of the human tumor-associated isozymes IX and XII with amino acids and amines. Bioorg Med Chem 2008;16:3530–6. (b) Innocenti A, Hilvo M, Parkkila S, et al. Carbonic anhydrase activators: Activation of the membrane-associated isoform XV with amino acids and amines. Bioorg Med Chem Lett 2009;19:3430–3. 10.1016/j.bmc.2008.02.021 18294854

[30] Nguyen GTH, Tran TN, Podgorski MN, et al. Nanoscale ion emitters in native mass spectrometry for measuring Ligand-Protein binding affinities. ACS Cent Sci 2019;5:308–18. 30834319 10.1021/acscentsci.8b00787 PMC6396573

[31] Innocenti A, Zimmerman SA, Scozzafava A, et al. Carbonic anhydrase activators: Activation of the archaeal β-class (Cab) and γ-class (Cam) carbonic anhydrases with amino acids and amines. Bioorg Med Chem Lett 2008;18:6194–8. 18930395 10.1016/j.bmcl.2008.10.005

[32] Vullo D, Del Prete S, Capasso C, Supuran CT Carbonic anhydrase activators: Activation of the β-carbonic anhydrase from *Malassezia globosa* with amino acids and amines. Bioorg Med Chem Lett 2016;26:1381–5. 26856923 10.1016/j.bmcl.2016.01.078

[33] Isik S, Kockar F, Aydin M, et al. Carbonic anhydrase activators: Activation of the β-carbonic anhydrase Nce103 from the yeast *Saccharomyces cerevisiae* with amino acids and amines. Bioorg Med Chem Lett 2009;19:1662–5. 19231177 10.1016/j.bmcl.2009.01.105

[34] Innocenti A, Leewattanapasuk W, Manole G, et al. Carbonic anhydrase activators: Activation of the β-carbonic anhydrase from the pathogenic yeast *Candida glabrata* with amino acids and amines. Bioorg Med Chem Lett 2010;20:1701–4. 20129782 10.1016/j.bmcl.2010.01.054

[35] (a) Angeli A, Del Prete S, Osman SM, et al. Activation studies of the α- and β-carbonic anhydrases from the pathogenic bacterium Vibrio cholerae with amines and amino acids. J Enzyme Inhib Med Chem 2018;33:227–33. (b) Angeli A, Del Prete S, Donald WA, et al. The γ-carbonic anhydrase from the pathogenic bacterium *Vibrio cholerae* is potently activated by amines and amino acids. Bioorg Chem 2018;77:1–5. 10.1080/14756366.2017.1412316 PMC7012002 29231751

[36] Angeli A, Del Prete S, Osman SM, et al. Activation studies with amines and amino acids of the β-carbonic anhydrase encoded by the Rv3273 gene from the pathogenic bacterium *Mycobacterium tuberculosis*. J Enzyme Inhib Med Chem 2018;33:364–9. 29322836 10.1080/14756366.2017.1422250 PMC6009870

[37] (a) Angeli A, Del Prete S, Pinteala M, et al. The first activation study of the β-carbonic anhydrases from the pathogenic bacteria Brucella suis and Francisella tularensis with amines and amino acids. J Enzyme Inhib Med Chem 2019;34:1178–85. (b) Stefanucci A, Angeli A, Dimmito MP, et al. Activation of β- and γ-carbonic anhydrases from pathogenic bacteria with tripeptides. J Enzyme Inhib Med Chem 2018;33:945–50. 10.1080/14756366.2019.1630617 PMC6691884 31282230

[38] Bua S, Haapanen S, Kuuslahti M, et al. Activation studies of the β-carbonic anhydrase from the pathogenic protozoan *Entamoeba histolytica* with amino acids and amines. Metabolites 2019;9:26–33. 10.3390/metabo9020026 PMC6409850 30717275

[39] Angeli A, Donald WA, Parkkila S, Supuran CT Activation studies with amines and amino acids of the β-carbonic anhydrase from the pathogenic protozoan *Leishmania donovani chagasi*. Bioorg. Chem 2018;78:406–10. 29689418 10.1016/j.bioorg.2018.04.010

[40] (a) Vullo D, Del Prete S, Osman SM, et al. *Burkholderia pseudomallei* γ-carbonic anhydrase is strongly activated by amino acids and amines. Bioorg Med Chem Lett 2017;27:77–80. (b) Vullo D, Del Prete S, Osman SM, et al. Comparison of the amine/amino acid activation profiles of the β- and γ-carbonic anhydrases from the pathogenic bacterium Burkholderia pseudomallei. J Enzyme Inhib Med Chem 2018;33:25–30. 10.1016/j.bmcl.2016.11.027 27881231

[41] Angeli A, Del Prete S, Osman SM, et al. Activation studies of the γ-carbonic anhydrases from the Antarctic marine bacteria Pseudoalteromonas haloplanktis and Colwellia psychrerythraea with amino acids and amines. Marine Drugs 2019;17:238–46. 10.3390/md17040238 PMC6520686 31013612

[42] Angeli A, Alasmary FAS, Del Prete S, et al. The first study of a δ-carbonic anhydrase: TweCAδ from the diatom Thalassiosira weissflogii is effectively activated by amines and amino acids. J Enzyme Inhib Med Chem 2018;33:680–5. 29536765 10.1080/14756366.2018.1447570 PMC6009927

[43] Angeli A, Buonanno M, Donald WA, et al The zinc - but not cadmium – containing ζ-carbonic from the diatom Thalassiosira weissflogii is potently activated by amines and amino acids . Bioorg Chem 2018;80:261–5. 29966872 10.1016/j.bioorg.2018.05.027

[44] Angeli A, Del Prete S, Alasmary FAS, et al. The first activation studies of the η-carbonic anhydrase from the malaria parasite Plasmodium falciparum with amines and amino acids. Bioorg Chem 2018;80:94–8. 29894892 10.1016/j.bioorg.2018.06.002

